# Brain–Behavior Coupling During Cognitive–Postural Dual‐Tasking in Older Adults With Mild Cognitive Impairment: An fNIRS Study

**DOI:** 10.1002/brb3.71907

**Published:** 2026-11-06

**Authors:** Chaojie Kan, Shizhe Zhu, Qinglei Wang, Junfan Shen, Sheng Xu, Yuefeng Li, Ren Zhuang, Chuan Guo

**Affiliations:** ^1^ Department of Rehabilitation Medicine Changzhou Dean Hospital Changzhou China; ^2^ Department of Rehabilitation Medicine The First Affiliated Hospital of Nanjing Medical University Nanjing China; ^3^ Jiangsu Key Laboratory of Medical Science and Laboratory Medicine, School of Medicine Jiangsu University Zhenjiang China

**Keywords:** brain–behavior coupling, dual task, mild cognitive impairment, postural control

## Abstract

**Background:**

Mild cognitive impairment (MCI) is associated with subtle deficits in cognitive‐motor integration and postural control; however, the cortical mechanisms underlying impaired cognitive–postural dual‐task performance remain unclear. This study aimed to investigate behavioral alterations, cortical activation patterns, and brain–behavior coupling characteristics during cognitive–postural dual‐tasking in older adults with MCI.

**Methods:**

Fifty‐three community‐dwelling older adults were enrolled, including 26 individuals with MCI and 27 cognitively normal controls. Participants performed a verbal fluency task while maintaining upright standing under four dual‐task conditions combining visual availability (eyes open/closed) and support‐surface stability (stable/unstable). Postural performance was assessed using the anteroposterior stability index (API) and mediolateral stability index (MLI), while cognitive performance was evaluated using verbal fluency scores. Functional near‐infrared spectroscopy (fNIRS) was used to measure task‐related cortical activation in bilateral prefrontal, primary somatosensory, primary motor, and premotor cortices. Between‐group comparisons, partial correlation analyses, and regression‐based interaction analyses were conducted to examine alterations in brain–behavior relationships.

**Results:**

Compared with cognitively normal controls, participants with MCI exhibited poorer postural stability under dual‐task conditions involving reduced visual input and/or unstable support surfaces. Reduced cortical activation was observed mainly in prefrontal regions during unstable support with eyes open and extended to sensorimotor‐related regions under combined visual deprivation and unstable support. Brain–behavior associations differed between groups. In cognitively normal older adults, increased cortical activation was generally associated with better behavioral performance. In contrast, individuals with MCI showed stronger associations between reduced activation of distributed prefrontal–sensorimotor regions and impaired postural performance, particularly under the eyes‐closed unstable‐support condition. Regression‐based interaction analysis further demonstrated a significant group‐dependent association between right prefrontal activation and mediolateral postural stability.

**Conclusions:**

Older adults with MCI exhibit altered brain–behavior coupling during cognitive–postural dual‐task conditions involving sensory and postural constraints, characterized by altered relationships between prefrontal–sensorimotor cortical activation and behavioral performance. These findings provide new insights into the neural mechanisms underlying cognitive‐motor dysfunction in MCI and highlight abnormal brain–behavior coupling as a potential feature of early functional impairment.

## Graphical abstract

Among 53 older adults, mild cognitive impairment was associated with poorer balance and reduced cortical activation under challenging cognitive‐postural dual tasks. Under eyes‐closed, unstable‐surface conditions, right prefrontal activation showed a stronger association with mediolateral sway in the mild cognitive impairment group than in controls (FDR‐adjusted *p* < 0.001).

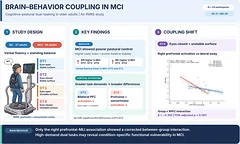

## 1 Introduction

Mild cognitive impairment (MCI) is widely regarded as an intermediate stage between normal aging and dementia, but its clinical significance extends beyond cognitive decline alone (Ramírez and Gutiérrez 2021). Increasing evidence suggests that older adults with MCI may also exhibit subtle impairments in motor function, postural regulation, and the ability to manage simultaneous cognitive and motor demands, all of which are closely related to falls, loss of independence, and reduced quality of life (G. Xu, Zhou, Chen, et al. 2024; Pitts et al. 2025). In real‐world settings, maintaining an upright balance rarely occurs in isolation; instead, it typically involves concurrent cognitive processing, such as speaking, planning, monitoring the environment, or responding to external cues. For this reason, cognitive–postural dual‐task paradigms have become an important framework for understanding functional vulnerability in aging and cognitive impairment (Wang et al. 2023). Recent reviews further indicate that balance dysfunction and dual‐task interference may represent clinically meaningful and potentially modifiable features in older adults with cognitive decline (Zeng et al. 2025).

A previous study has shown that older adults with MCI perform worse than cognitively normal older adults under dual‐task conditions, including increased postural sway, poorer mobility, and greater cognitive‐motor interference (Bahureksa et al. 2017). At the neural level, functional near‐infrared spectroscopy (fNIRS) has become a valuable tool for investigating these processes because it allows the assessment of cortical hemodynamic activity during ecologically relevant tasks such as standing and walking (Kvist et al. 2023). Existing fNIRS studies have consistently implicated the prefrontal cortex (PFC) in attentional allocation and executive control during dual‐task performance (Udina et al. 2020). In particular, G. Xu, Zhou, Chen, et al. (2024) demonstrated that older adults with MCI showed poorer standing balance and altered prefrontal oxygenation during balance‐related dual tasks, supporting the idea that impaired cortical regulation contributes to functional decline in this population. Meanwhile, recent work has also emphasized that the neural basis of dual‐task control is unlikely to be limited to the PFC alone but may involve broader cortical systems related to sensorimotor integration, motor planning, and network efficiency (Ding et al. 2024). In addition, a recent meta‐analysis reported that verbal fluency tasks produce relatively strong age‐related cognitive‐motor interference, suggesting that they may be especially suitable for probing resource competition during dual‐task performance in older adults (Harrison et al. 2025).

Despite these advances, several important gaps remain. First, previous studies in MCI have more often focused on gait or relatively simple balance tasks, whereas less is known about how MCI affects standing cognitive–postural dual‐task performance under systematically graded sensory and support‐surface challenges (Bahureksa et al. 2017). Second, although prior fNIRS research has highlighted prefrontal involvement, balance control under dual‐task conditions is likely supported by a distributed cortical network that also includes the primary somatosensory, primary motor, and premotor regions; these areas have been less comprehensively examined in MCI (Krougly et al. 2025). Third, the relationship between cortical activation and behavioral performance remains insufficiently understood (Lehmann et al. 2022). Recent evidence suggests that brain–behavior associations in standing balance are influenced by task characteristics and participant status, but whether such associations emerge only under specific combinations of sensory and postural demands and whether their patterns differ fundamentally between cognitively normal older adults and those with MCI (G. Xu, Zhou, Chen, et al. 2024, Stuart et al. 2018). Therefore, the field still lacks a more integrated account of when dual‐task deficits become apparent in MCI, which cortical regions are most affected as task demands increase, and how cortical recruitment relates to behavioral performance across different cognitive states.

To address these issues, the present study aimed to investigate behavioral performance and cortical activation during cognitive–postural dual‐tasking in older adults with MCI and cognitively normal older adults, using a verbal fluency‐based standing balance paradigm involving different combinations of visual availability and support‐surface stability. More specifically, this study sought to address three closely related scientific questions: whether older adults with MCI exhibit poorer balance and cognitive performance under increasingly demanding dual‐task conditions; whether these behavioral deficits are accompanied by reduced activation in prefrontal and sensorimotor‐related cortical regions; and whether the relationship between cortical activation and behavioral performance differs between cognitively normal and MCI groups across different sensory and postural conditions.

To address these issues, we designed four dual‐task conditions by combining two visual states (eyes open and eyes closed) with two support‐surface conditions (stable and unstable), thereby creating four combinations of visual availability and support‐surface stability. Balance performance was quantified using the anteroposterior stability index (API) and mediolateral stability index (MLI), cognitive performance was assessed using verbal fluency test (VFT) scores, and cortical activation was measured with fNIRS across eight regions of interest covering the bilateral prefrontal, primary somatosensory, primary motor, and premotor cortices. In addition to between‐group comparisons, partial correlation analyses were conducted separately in the two groups to examine associations between behavioral outcomes and cortical activation. This study has three main strengths: first, it applies a condition‐specific standing dual‐task paradigm incorporating different combinations of sensory and postural demands that is sensitive to changes in sensory and postural demands; second, it extends cortical assessment beyond the prefrontal cortex to include key sensorimotor regions involved in balance regulation; and third, it combines behavioral and brain–behavior coupling analyses to provide a more integrated characterization of dual‐task dysfunction in MCI. On the basis of prior findings and the present design, we hypothesized that older adults with MCI would show poorer dual‐task performance, reduced activation in prefrontal and sensorimotor‐related regions, and altered brain–behavior coupling, particularly under conditions involving reduced visual availability and/or unstable support surfaces.

## 2 Methods

### 2.1 Participants

Community‐dwelling older adults aged 60 years or above were recruited between August and September 2025 for this study. Participants’ cognitive function was assessed across three dimensions: memory, executive functioning, and language ability. Episodic memory was measured using the long‐delay recall and recognition components of the auditory verbal learning test (AVLT). Executive functioning was assessed with the shape trail test, including both Part A (STT‐A) and Part B (STT‐B). Language ability was evaluated using the Boston naming test (BNT) together with the VFT. MCI was diagnosed using the comprehensive neuropsychological criteria reported by Polcher et al. (2022). Specifically, participants were classified as having MCI if they showed either two impaired neuropsychological test scores (>1.0 SD below the normative mean) within at least one cognitive domain, or one impaired test score (>1.0 SD below the normative mean) in each of the three cognitive domains evaluated. Older adults who did not meet these criteria were assigned to the normal cognition (NC) group. All enrolled participants were right‐handed. Participants were excluded if they had (1) a prior history of central nervous system disorders, (2) impairments involving the visual, vestibular, or proprioceptive systems, (3) medical conditions that could compromise sensory or motor function of the lower limbs, or (4) a history of receiving drug‐based or nondrug interventions targeting cognitive impairment.

Sample size estimation was performed using G·Power version 3.1. Based on findings from previous studies, the effect size was estimated from the between‐group difference in the relevant outcome measure, and Cohen's *d* was set at 0.81 (G. Xu, Zhou, Chen, et al. 2024). The sample size calculation was conducted for a two‐sided comparison of two independent means, with a significance level of *α* = 0.05 and a statistical power of 80% (1 ‐ *β*= 0.80). The results indicated that at least 25 participants were required in each group, corresponding to a minimum total sample size of 50. Finally, 53 older adults were enrolled in the present study, including 27 in the NC group (11 men and 16 women) and 26 in the MCI group (11 men and 15 women), which satisfied the sample size requirement. Other baseline characteristics are shown in Table 1.

**TABLE 1:** Baseline characteristics.

	NC group (*n = *27)	MCI group (*n = *26)	*t*/*Z*	*p*
Age	65.000(64.000, 71.000)	67.000(63.000, 70.000)	−0.187^a^	0.851
BMI	24.311 ± 3.135	23.773±2.067	0.740	0.463
Educational level	3(2, 3)	3(2, 3)	−0.917^a^	0.359
AVLT: Long‐delayed Recall	4.000(4.000, 5.000)	4.000(2.000, 5.000)	−1.294^a^	0.196
AVLT: Recognition	21(20, 22)	19.500(16.750, 21.000)	−3.332^a^	0.001
STT‐A	58.000(52.000, 67.000)	98.500(75.250, 159.500)	−4.111^a^	< 0.001
STT‐B	174.222 ± 55.890	278.346 ± 84.533	−5.309	< 0.001
BNT	21.741 ± 3.206	16.654 ± 4.260	4.924	< 0.001
VFT	16.778 ± 2.501	12.346 ± 1.938	7.191	< 0.001

^a^ *Z* value.

^b^ Education level: illiterate = 1, primary school = 2, junior high school = 3, senior high school = 4, and college or above = 5.

This study was conducted in accordance with the Declaration of Helsinki. Approval was granted by the Ethics Committee of Changzhou Dean Hospital (No. CZDALL‐2025‐002), and the study was registered with the Chinese Clinical Trial Registry (No. ChiCTR2500107603). All participants provided written informed consent before participation.

### 2.2 Dual‐Task Procedure

In the dual‐task paradigm, the cognitive task was the VFT, in which participants were asked to produce as many words as possible within a fixed time interval from a designated semantic category, including vegetables, fruits, animals, or modes of transportation. Under dual‐task conditions, the VFT has been shown to be sensitive to cognitive alterations in older adults with MCI (Harrison et al. 2025). The postural control task involved maintaining an upright standing position under four combinations created by two visual conditions (eyes open and eyes closed) and two support‐surface conditions (stable and unstable). Based on these combinations, four dual‐task conditions were established: (1) DT1, standing on a stable surface with eyes open while completing the VFT; (2) DT2, standing on a stable surface with eyes closed while completing the VFT; (3) DT3, standing on an unstable surface with eyes open while completing the VFT; and (4) DT4, standing on an unstable surface with eyes closed while completing the VFT.

Prior to the experiment, all participants were equipped with the fNIRS cap. They were instructed to face forward, relax both arms alongside the body, stand comfortably on the balance platform with feet placed naturally apart, and wait for the researcher's cue to begin. The balance platform was capable of generating either a stable or an unstable support surface. Baseline fNIRS signals were collected for 10 s before the onset of the task. Thereafter, prerecorded verbal instructions delivered by computer were presented at predetermined time points, including “close your eyes,” “maintain balance,” “please name fruits/vegetables/animals/means of transportation,” and “rest.” Participants were required to initiate the corresponding dual‐task condition immediately after each instruction was given.

The order of the four dual‐task conditions was randomized across participants to minimize potential order effects. For the unstable condition, the Biodex balance system was set at stability level 12, representing the least unstable level among the 12 available stability settings. Before each trial, participants adopted a comfortable standing posture on the platform, and individual foot placement was maintained consistently across all experimental conditions.

A block design was adopted for each dual‐task condition, and each condition was performed twice, yielding a total duration of 120 s. Each block lasted 60 s and consisted of a 30‐s task phase, during which participants carried out the dual task, followed by a 30‐s rest phase, during which they resumed the initial standing posture. The experimental procedure is illustrated in Figure 1. Across all testing conditions, participants were asked to maintain an upright posture as steadily as possible. Before the formal assessment, sufficient time was provided for all participants to familiarize themselves with the protocol. During the testing session, a researcher remained behind each participant to ensure safety. The experimental setup is illustrated in Figure 2.

**FIGURE 1:**
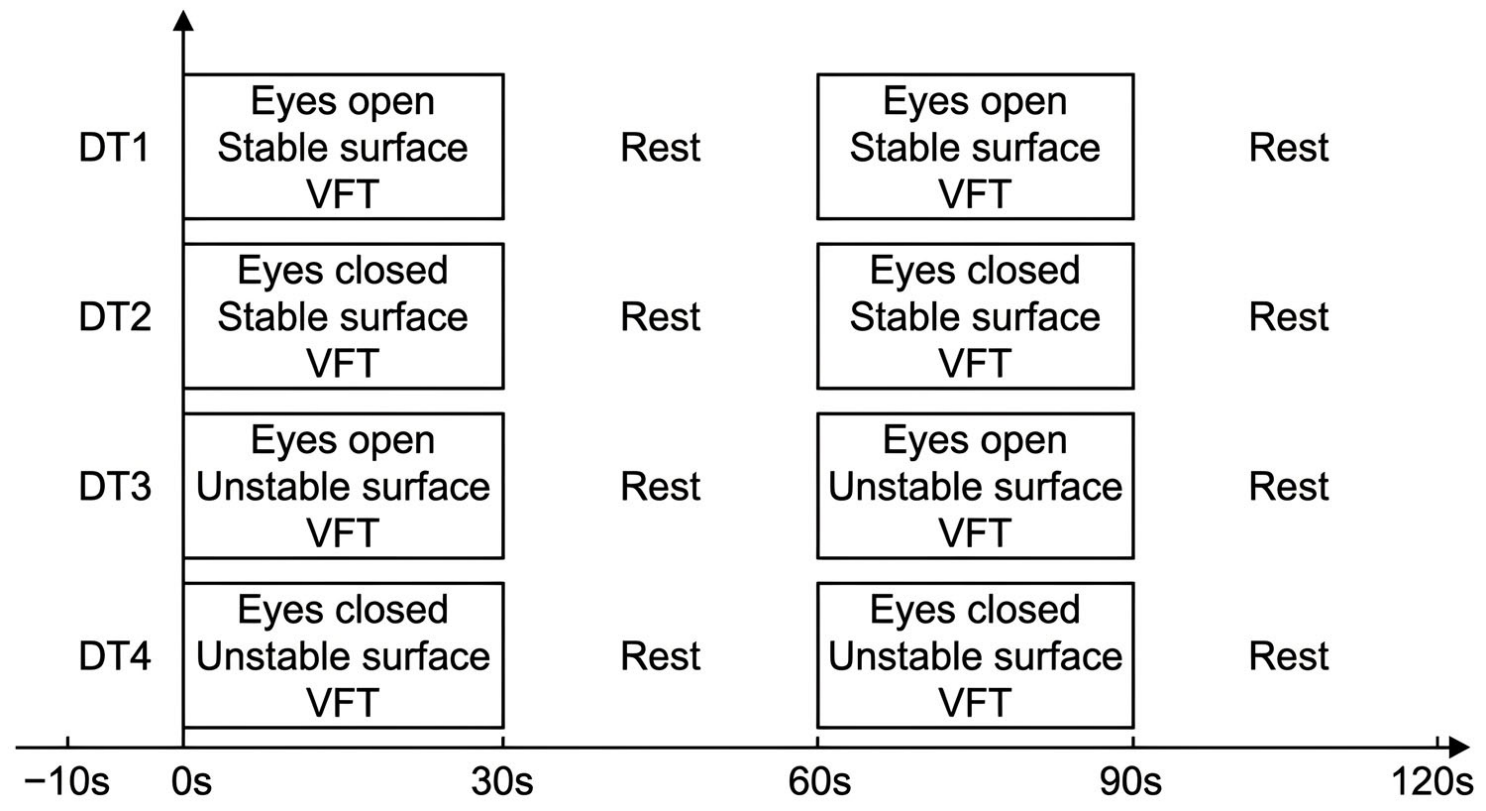
Experimental paradigm of the dual‐task procedure. Each condition consisted of two 30‐s task blocks interleaved with two 30‐s rest blocks, yielding a total duration of 120 s. DT1, standing on a stable surface with eyes open while performing the VFT; DT2, standing on a stable surface with eyes closed while performing the VFT; DT3, standing on an unstable surface with eyes open while performing the VFT; and DT4, standing on an unstable surface with eyes closed while performing the VFT.

**FIGURE 2:**
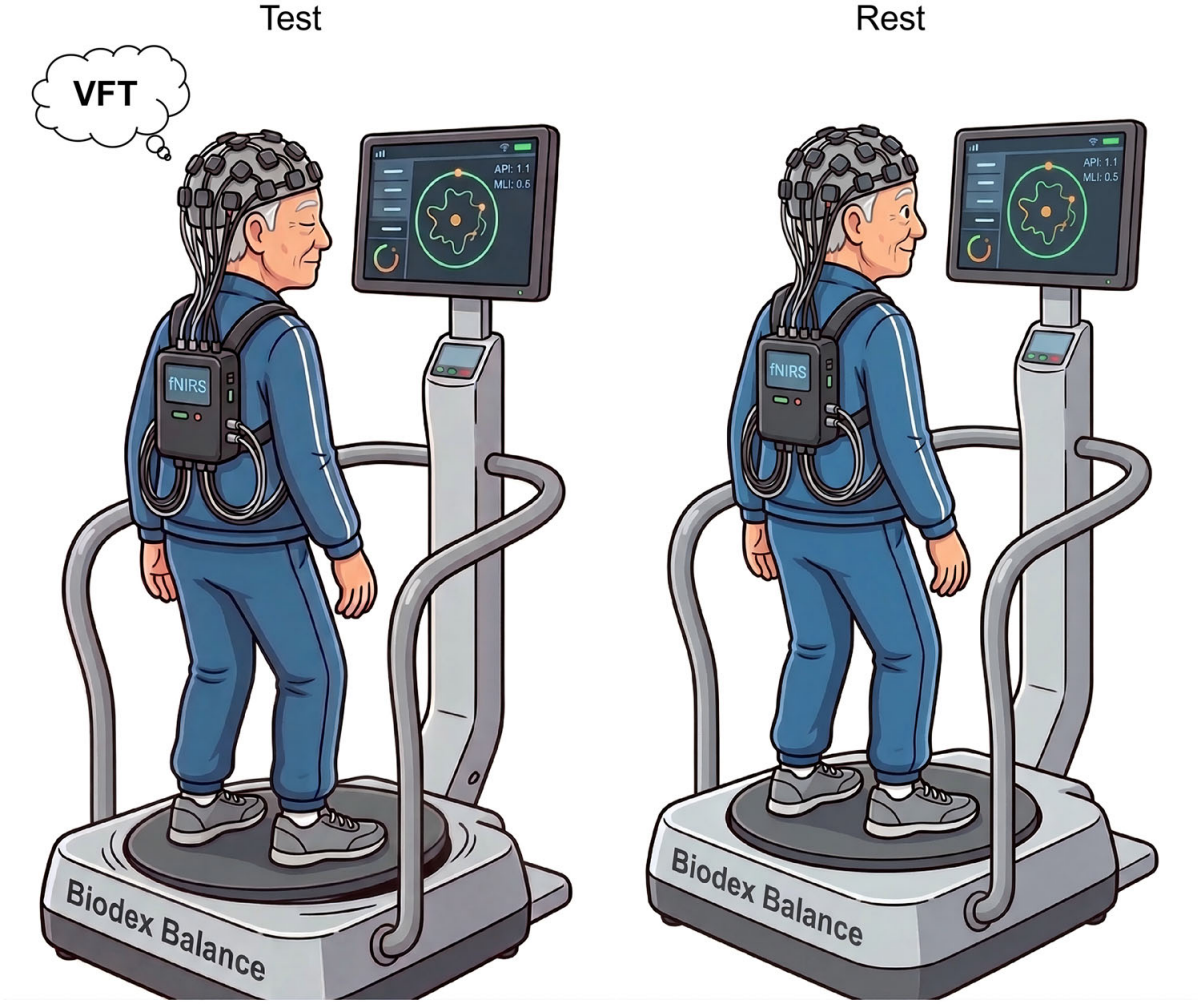
The experimental setup. Participants wore an fNIRS cap while standing on the balance platform and performed dual‐task trials during the test phase. During the rest phase, participants maintained a relaxed standing posture on the platform with their feet comfortably apart, arms relaxed at their sides, and gaze directed forward.

### 2.3 Behavioral Data Collection

#### 2.3.1 Balance Outcome

Balance performance was assessed using the API and MLI obtained from a computerized balance platform (Biodex Medical Systems, Shirley, NY, USA). The platform continuously records postural sway as angular deviations from the horizontal plane in the anteroposterior (AP) and mediolateral (ML) directions. API and MLI were used to characterize directional postural sway in the AP and ML planes, respectively, with higher values indicating poorer balance stability.

#### 2.3.2 Cognitive Outcome

Performance on the cognitive task was evaluated using the VFT scores. In each 30‐s task phase, participants were asked to generate words belonging to a specified semantic category, such as vegetables, fruits, animals, or means of transportation. These semantic categories were randomly assigned across task conditions and repetitions to minimize potential confounding effects related to category‐specific differences in retrieval difficulty. Each dual‐task condition included two task phases, and the total number of correctly named items across both phases was taken as the cognitive task score for that condition.

### 2.4 Cortical Activation Data Collection

Cortical hemodynamic activity was recorded using a fNIRS system (NirSmart‐3000A, Danyang Huichuang Medical Equipment Co. Ltd., China). The system consisted of 19 sources and 16 detectors, forming 46 measurement channels with an inter‐optode distance of 3 cm. Concentration changes in oxygenated hemoglobin (HbO) and deoxygenated hemoglobin (HbR) were measured at wavelengths of 730 and 850 nm, respectively, with a sampling rate of 11 Hz. Channel locations were registered in advance to a standard brain template using a three‐dimensional digitizing pen. Based on this spatial registration, each channel was assigned to the Brodmann area with the highest anatomical probability, which was defined as the corresponding cortical region for that channel.

In fNIRS studies involving dual‐task paradigms, regions of interest (ROIs) are commonly selected from cortical areas associated with cognitive and motor processing. Accordingly, the ROIs in the present study included the right prefrontal cortex (RPFC), right primary somatosensory cortex (RS1), right primary motor cortex (RM1), right premotor cortex (RPMC), left prefrontal cortex (LPFC), left primary somatosensory cortex (LS1), left primary motor cortex (LM1), and left premotor cortex (LPMC). The fNIRS channel layout and ROI distribution are shown in Figure 3.

**FIGURE 3:**
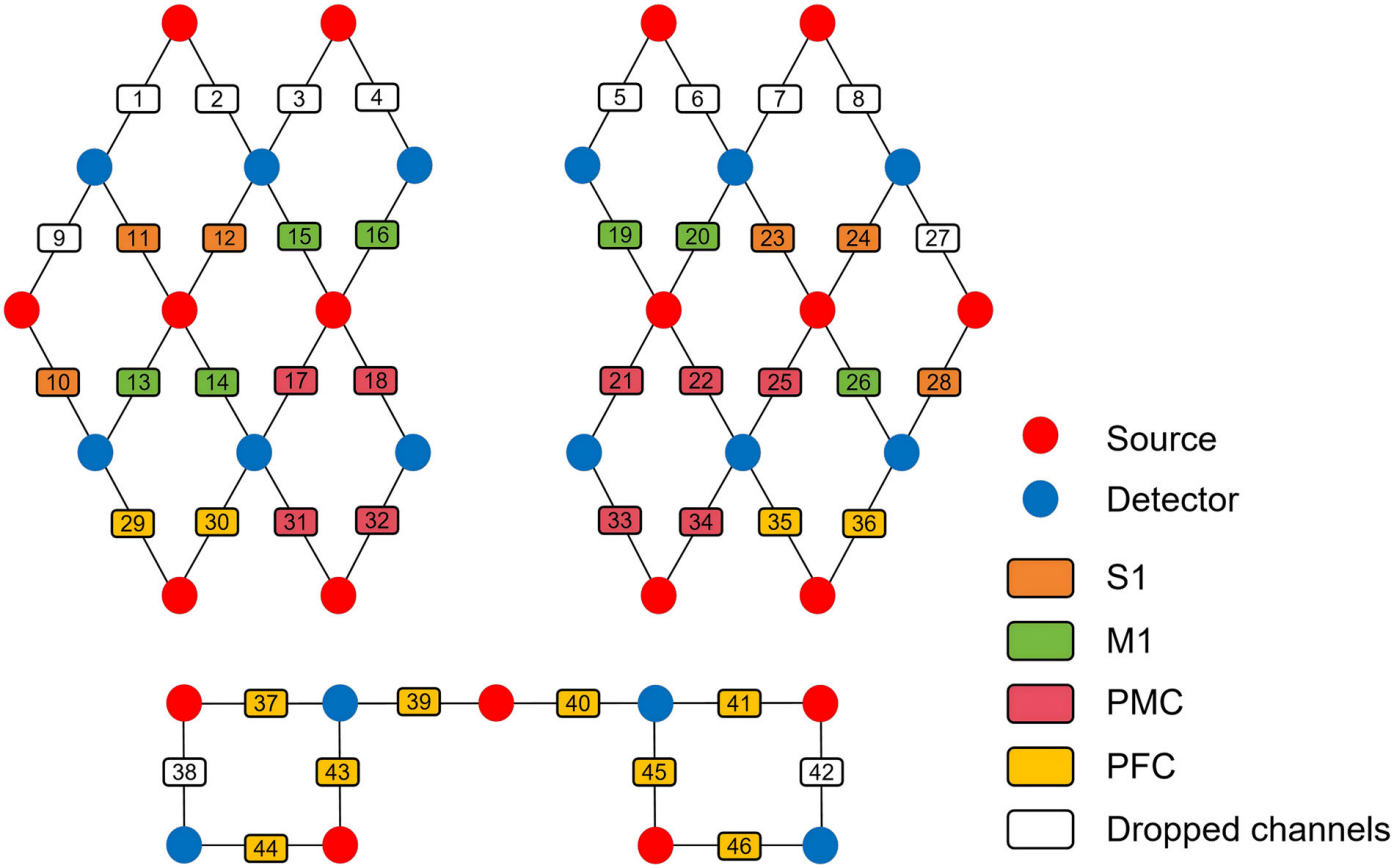
fNIRS channel configuration and ROI map. S1, primary somatosensory cortex; M1, primary motor cortex; PMC, the premotor cortex; and PFC, the prefrontal cortex.

Cortical hemodynamic data were processed using NirSpark software. During preprocessing, motion artifacts were corrected by spline interpolation, with the standard deviation threshold set to 6 and the amplitude threshold set to 0.5. A band‐pass filter of 0.01–0.1 Hz was then applied to reduce physiological noise and low‐frequency drift.

A block‐averaging approach was used for task‐related analysis. Task onset was defined as 0 s, the baseline period was set from −10 to 0 s, and the subsequent 30 s was used as the analysis window to obtain the mean hemodynamic response. The block‐averaged waveform was used to represent the average cortical hemodynamic change evoked by the task.

To further quantify task‐related cortical activation, a general linear model (GLM) was fitted to the preprocessed hemoglobin concentration time series:

Y(t)=X(t)β+ε

where *Y(t)* represents the hemoglobin concentration time series, *X(t)* denotes the design matrix for the task condition, and *β* is the regression coefficient reflecting the magnitude of the task‐evoked hemodynamic response. The *β* value was taken as an index of cortical activation intensity.

### 2.5 Statistical Analysis

All statistical analyses were performed using SPSS version 25.0 (IBM Corp., Armonk, NY, USA). Behavioral measures, including the API, MLI, and VFT scores, as well as cortical activation (*β* values) in the eight ROIs, were compared between the NC and MCI groups under each dual‐task condition. Because the primary aim of this study was to characterize condition‐specific behavioral and cortical alterations associated with MCI, rather than to estimate factorial interaction effects among visual and surface manipulations, between‐group comparisons were conducted separately for each dual‐task condition. For variables that were normally distributed, between‐group comparisons were performed using the independent‐samples *t* test, and the data are presented as ($\bar{x}$ *± s*). For variables that were not normally distributed, between‐group comparisons were conducted using the Mann–Whitney U test, and the data are presented as *M*(*Q1, Q3*).

In addition, partial correlation analyses were performed separately in the NC and MCI groups to examine the associations between behavioral measures (API, MLI, and VFT) and cortical activation in the eight ROIs, while controlling for age and years of education. For variables that met the assumption of normality, Pearson partial correlation analysis was used. For variables that were not normally distributed, rank transformation was performed at the individual‐case level prior to partial correlation analysis, and the correlations were then estimated based on the ranked data to reduce the influence of non‐normality.

To directly test whether brain–behavior associations differed between the NC and MCI groups, additional exploratory regression‐based interaction analyses were performed for selected brain–behavior associations identified from the within‐group partial correlation analyses. For each model, behavioral performance was entered as the dependent variable, and cortical activation, group, and the cortical activation × group interaction term were entered as predictors, with age and education included as covariates.

To account for multiple comparisons across the eight ROIs, the *p* values obtained from both the between‐group comparisons and partial correlation analyses were further corrected using the false discovery rate (FDR) method, and the corrected *p* values are reported as *p*_adj_. A two‐tailed *p* < 0.05 was considered statistically significant, and for FDR‐corrected analyses, *p*_adj_ < 0.05 was considered statistically significant.

## 3 Results

### 3.1 Between‐Group Comparisons

#### 3.1.1 Behavioral Performance Under Dual‐Task Conditions

Significant between‐group differences were observed in behavioral performance under dual‐task conditions and the full results are provided in Table S1 at the end of the manuscript. For balance performance, differences were identified in both the API and MLI under DT2–DT4. Specifically, the MCI group showed significantly higher API values than the NC group in DT2 (*Z* = −1.997, *p* = 0.046), DT3 (*Z* = −2.075, *p* = 0.038), and DT4 (*Z* = −2.297, *p* = 0.022), whereas no significant between‐group difference was found in DT1 (*p* > 0.05). For MLI, significantly higher values were observed in the MCI group in DT2 (*Z* = −2.304, *p* = 0.021) and DT4 (*Z* = −2.915, *p* = 0.004), while no significant differences were found in DT1 or DT3 (*p* > 0.05). The balance‐related results are shown in Figure 4A,B. These results suggest that older adults with MCI exhibited poorer postural control than cognitively normal older adults, particularly under conditions involving reduced visual input and/or unstable support surfaces.

**FIGURE 4:**
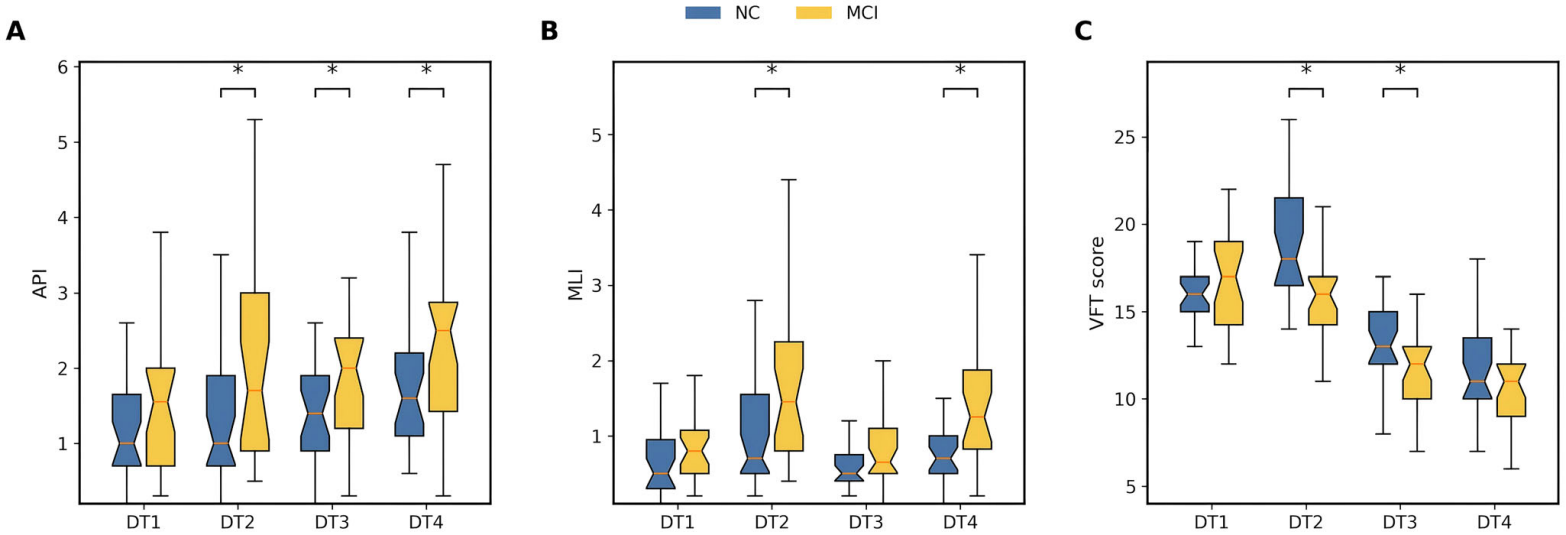
Group differences in API, MLI, and VFT across task stages. Boxplots showing the distributions of API (A), MLI (B), and VFT score (C) across four task stages (DT1–DT4) in the NC and MCI groups. The central line represents the median, the box indicates the interquartile range (IQR), and the whiskers extend to the fifth and 95th percentiles. Notches represent the approximate 95% confidence interval of the median. Significant between‐group differences at each task stage are indicated by asterisks (*, *p* < 0.05).

For VFT performance, the MCI group scored significantly lower than the NC group in DT2 (*t* = 3.643, *p* = 0.001) and DT3 (*t* = 2.729, *p* = 0.009), whereas no significant between‐group differences were observed in DT1 or DT4 (*p* > 0.05). The cognitive results are shown in Figure 4C. These findings indicate that cognitive performance under dual‐task conditions was impaired in the MCI group, particularly under tasks with moderate balance demands.

#### 3.1.2 Between‐Group Differences in Cortical Activation

Significant between‐group differences in cortical activation were observed mainly in the prefrontal and sensorimotor‐related regions under DT3 and DT4. In DT3, the MCI group showed significantly lower activation than the NC group in the bilateral prefrontal cortex (RPFC: *Z* = −2.936, *p*_adj_ = 0.012; LPFC: *t* = 3.173, *p*_adj_ = 0.012). In DT4, significantly reduced β values were observed in the MCI group in the RPFC (*t = 3.888*, *p*_adj_ = 0.008), RS1 (*t* = 3.016, *p*_adj_ = 0.011), RM1 (*t* = 2.404, *p*_adj_ = 0.027), RPMC (*Z* = −2.900, *p*_adj_ = 0.011), LPFC (*Z* = −2.669, *p*_adj_ = 0.013), and LM1 (*t* = 2.825, *p*_adj_ = 0.013). No significant between‐group differences were detected in other regions under DT1 or DT2 (*p* > 0.05). Figure 5 further illustrates the between‐group differences in cortical activation across these conditions and the full results are provided in Table S2 at the end of the manuscript. These findings indicate that MCI was associated with reduced cortical activation during dual‐task performance, especially under conditions combining greater sensory and postural constraints.

**FIGURE 5:**
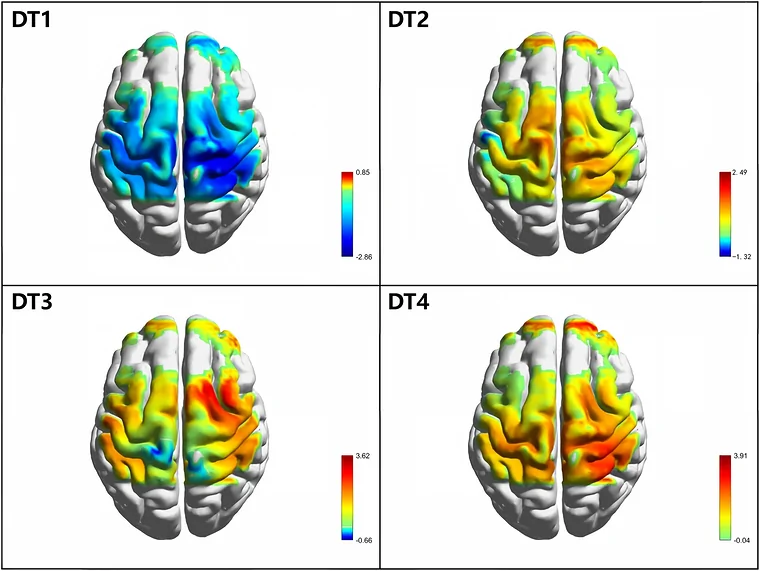
Between‐group differences in cortical activation under the four dual‐task conditions. The figure presents a descriptive channel‐wise visualization generated from independent‐samples *t*‐statistic maps of between‐group *β*‐value differences. The color scale represents the direction and magnitude of the t statistics for visualization purposes. Warm colors indicate higher cortical activation in the MCI group relative to the NC group, whereas cool colors indicate lower cortical activation in the MCI group. Formal statistical inference was performed at the ROI level using either independent‐samples *t* tests or Mann–Whitney U tests according to data distribution, as reported in Table S2.

### 3.2 Partial Correlation Analyses Between Behavioral Performance and Cortical Activation

#### 3.2.1 Partial Correlation Results in the NC Group

In the NC group, significant associations were observed primarily under DT3 and DT4 after FDR correction. During DT3, API was negatively correlated with activation in the RPFC, RS1, RPMC, LPFC, LS1, and LM1 (all *p*_adj_ < 0.05), whereas VFT scores were positively correlated with activation in the RPFC and LPFC (*p*_adj_ = 0.028 and 0.016, respectively). During DT4, MLI was negatively correlated with activation in the RPFC, LPFC, LS1, LM1, and LPMC (all *p*_adj_ < 0.05). No significant correlations were identified in DT1 or DT2 after FDR correction. These findings suggest that, in cognitively normal older adults, brain–behavior associations became evident mainly under relatively demanding dual‐task conditions. The partial correlation results for the NC group are shown in Figure 6 and the full results are provided in Table S3 at the end of the manuscript.

**FIGURE 6:**
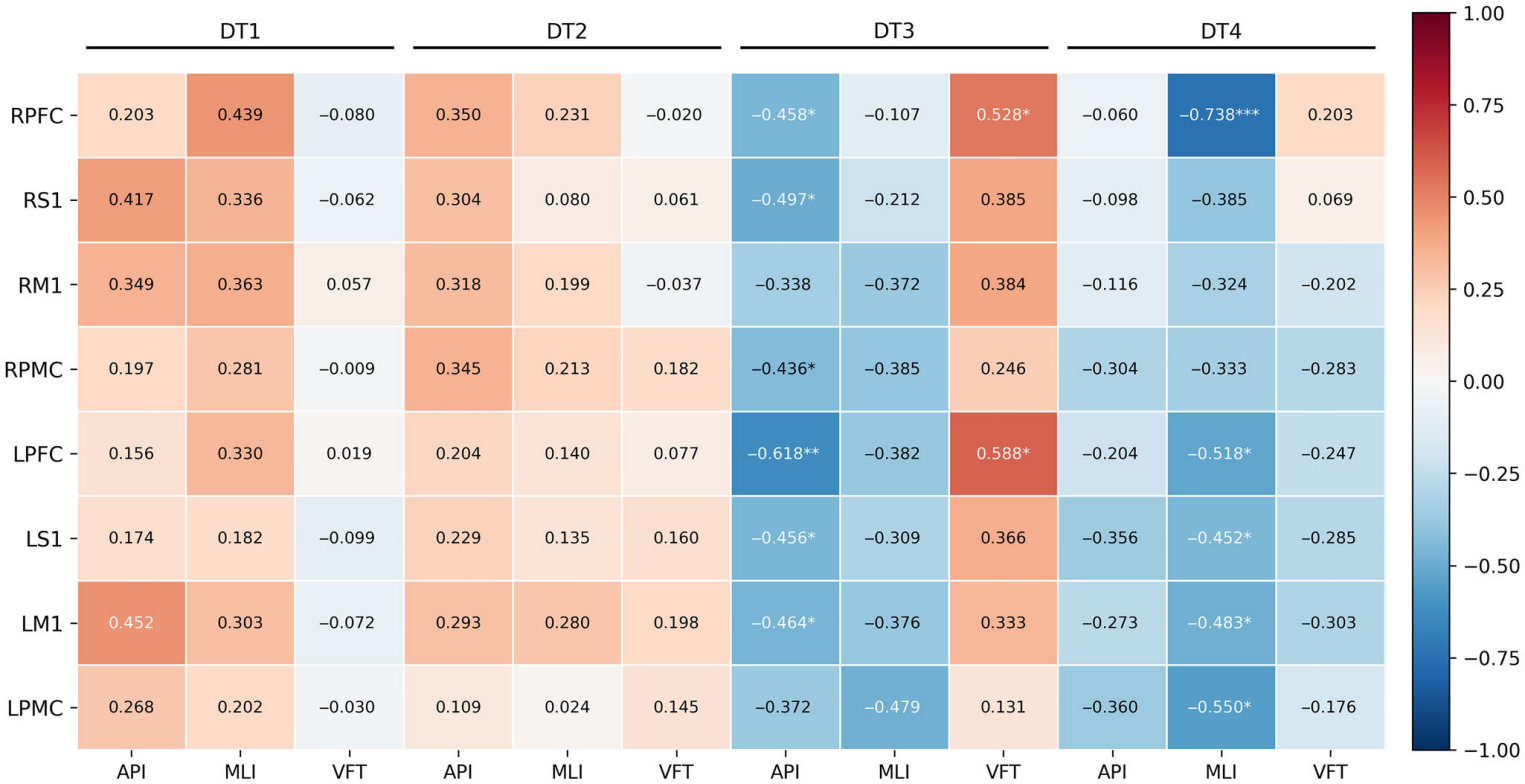
Heatmap of partial correlations between cortical activation and behavioral performance in the NC group during four dual‐task conditions. The heatmap displays the partial correlation coefficients between cortical activation in each region of interest and behavioral measures, including API, MLI, and VFT, across four dual‐task conditions (DT1–DT4) in the NC group. These partial correlations were calculated while controlling for age and years of education. The color bar represents the correlation coefficient (*r*), ranging from −1 to 1. Each cell is annotated with the corresponding r value. Statistical significance based on adjusted *p* values is indicated as follows: *p*_adj_ < 0.05, *; *p*_adj_ < 0.01, **; and *p*_adj_ < 0.001, ***.

#### 3.2.2 Partial Correlation Results in the MCI Group

In the MCI group, significant correlations were also mainly observed under more demanding conditions. During DT2, VFT scores were negatively correlated with LS1 activation (*p*_adj_ = 0.048). During DT3, VFT scores were positively correlated with activation in the RPFC and LPFC (both *p*_adj_ = 0.008). During DT4, API was negatively correlated with activation in the RPFC, RPMC, LPFC, LS1, LM1, and LPMC (all *p*_adj_ < 0.05), whereas MLI was negatively correlated with activation in the RPFC, RM1, RPMC, LPFC, LS1, LM1, and LPMC (all *p*_adj_ < 0.05). No other correlations remained significant after FDR correction. These findings suggest that, in older adults with MCI, poorer behavioral performance was closely associated with reduced activation in prefrontal and sensorimotor‐related regions, particularly under high‐demand dual‐task conditions. The partial correlation results for the MCI group are shown in Figure 7 and the full results are provided in Table S4 at the end of the manuscript.

**FIGURE 7:**
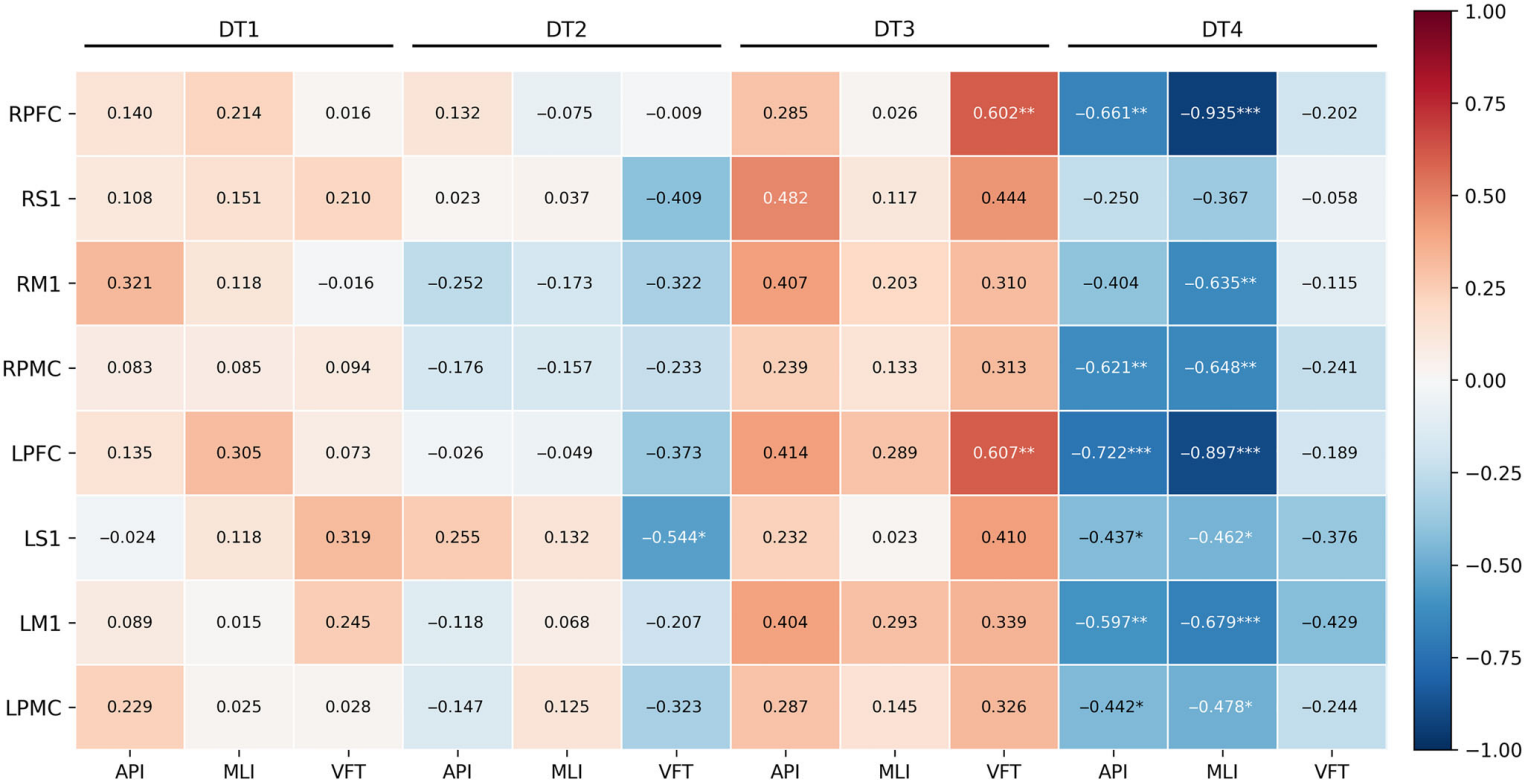
Heatmap of partial correlations between cortical activation and behavioral performance in the MCI group during four dual‐task conditions. The heatmap displays the partial correlation coefficients between cortical activation in each region of interest and behavioral measures, including API, MLI, and VFT, across four dual‐task conditions (DT1–DT4) in the MCI group. These partial correlations were computed after controlling for age and years of education. The color scale represents the correlation coefficient (*r*), ranging from −1 to 1. Each cell is annotated with the corresponding r value. Adjusted statistical significance is indicated as follows: *p*_adj_ < 0.05, *; *p*_adj_ < 0.01, **; and *p*_adj_ < 0.001, ***.

### 3.3 Direct Between‐Group Comparison of Brain–Behavior Coupling

To determine whether brain–behavior associations differed directly between groups, regression models including cortical activation × group interaction terms were performed. Based on the within‐group partial correlation analyses, three behavioral‐condition families were selected for exploratory between‐group interaction analyses: DT4–API, DT4–MLI, and DT3–VFT. These families were selected because they represented the statistically significant and interpretable brain–behavior associations observed within groups and covered both major functional domains assessed in this study, including postural control (API and MLI) and cognitive performance (VFT). After FDR correction across the eight ROIs within each hypothesis family, a significant group interaction was identified for the association between RPFC activation and MLI during DT4 (interaction β = −3.156, SE = 0.694, *t* = −4.547, 95% CI: −4.553 to −1.760, *p*_adj_ < 0.001). The estimated activation–MLI slope was −1.390 in the NC group and −4.546 in the MCI group, indicating a stronger negative association between RPFC activation and mediolateral postural sway in the MCI group under the eyes‐closed unstable‐support condition. The interaction pattern and group‐specific adjusted regression lines are illustrated in Figure 8.

**FIGURE 8:**
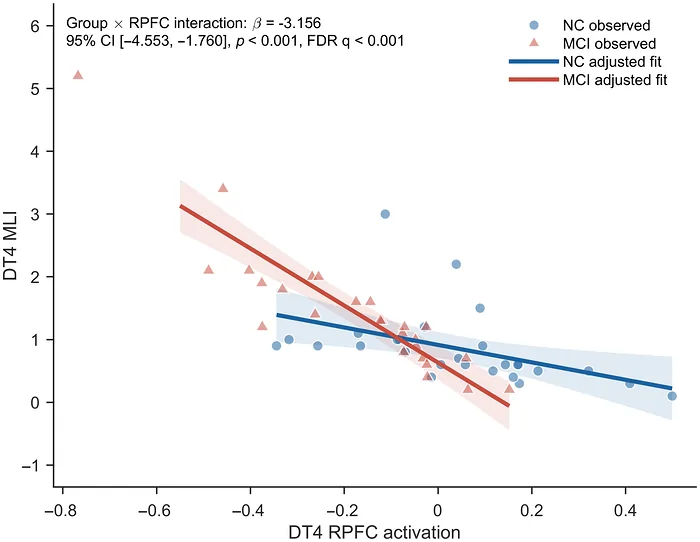
Group‐dependent association between RPFC activation and mediolateral postural stability during DT4. Scatter points represent individual observations from participants with NC and MCI. Solid lines represent model‐estimated regression lines adjusted for age and years of education, and shaded areas indicate the corresponding 95% confidence intervals of the fitted mean. A significant Group × RPFC interaction was observed for the association between RPFC activation and DT4 MLI after FDR correction (interaction *β* = −3.156, 95% CI: −4.553 to −1.760, *p*_adj_ < 0.001). The association between RPFC activation and MLI was significantly stronger in the MCI group than in the NC group, indicating altered brain–behavior coupling under the eyes‐closed unstable‐support condition.

For DT4–API, the RPFC activation × group interaction reached nominal significance but did not survive correction across the eight ROIs (*p*_adj_ = 0.062). No DT3 VFT interaction remained significant after FDR correction. Thus, among the exploratory interaction models tested, only the DT4 RPFC–MLI association remained significant after correction. Complete interaction results are provided in Table S5.

### 3.4 Sensitivity Analyses of the Large Correlations

Because particularly large partial correlations were observed between prefrontal activation and MLI in the MCI group during DT4, their robustness to influential observations was further examined. The RPFC–MLI partial correlation was *r* = −0.935 (95% CI: −0.972 to −0.854). Although two observations showed Cook's distance values exceeding the conventional 4/*n* reference value, leave‐one‐out analyses yielded partial correlations ranging from −0.962 to −0.921. After simultaneous exclusion of these observations, the association remained strong (*r* = −0.959).

Similarly, the LPFC‐MLI partial correlation was *r* = −0.897 (95% CI: −0.955 to −0.773). Leave‐one‐out estimates ranged from −0.917 to −0.881, and exclusion of the two observations with relatively elevated Cook's distance did not materially alter the association (*r* = −0.932). These sensitivity analyses indicate that the large prefrontal–MLI correlations were not driven by individual influential observations.

## 4 Discussion

The present study examined whether older adults with MCI show altered behavioral performance and cortical activation during cognitive–postural dual‐tasking under varying sensory and balance demands. Two principal findings emerged. First, compared with cognitively normal older adults, individuals with MCI demonstrated poorer dual‐task performance, as reflected by greater postural instability under conditions involving reduced visual input and/or unstable support surfaces and lower verbal fluency performance under selected conditions of moderate‐to‐high task demand. Second, these behavioral deficits were accompanied by reduced cortical activation, particularly in the bilateral prefrontal cortex under DT3 and in both prefrontal and sensorimotor‐related regions under DT4. In addition, significant brain–behavior associations were observed primarily under specific dual‐task conditions, particularly those combining reduced visual availability and unstable support surfaces, suggesting that the coupling between cortical activation and behavioral performance may depend on task‐specific sensory and postural demands.

A key finding of this study is that group differences were not evident across all task conditions, but emerged primarily under dual‐task conditions involving greater sensory and postural constraints. Under conditions with fewer sensory and postural constraints, behavioral differences between groups were limited, whereas clearer impairments emerged when visual input was reduced and/or postural stability was challenged (Qi et al. 2023, St George et al. 2021). This pattern suggests that older adults with MCI may still maintain relatively preserved performance under simpler dual‐task conditions, but that this adaptive capacity may become insufficient when visual availability is reduced and/or support‐surface stability is compromised. Such an interpretation is consistent with prior work showing that MCI is associated with impaired postural control, and that dual‐task standing in older adults is accompanied by altered frontal or broader cortical activity related to postural performance (Muir‐Hunter and Wittwer 2016; Manor et al. 2018; Ozdemir et al. 2016).

Another important observation is that between‐group differences in cortical activation were concentrated in the prefrontal and sensorimotor‐related regions under DT3 and DT4 conditions. Reduced activation in the bilateral prefrontal cortex was already detectable in DT3, whereas under DT4 this reduction extended to the primary somatosensory cortex, primary motor cortex, and premotor cortex. The involvement of additional sensorimotor‐related regions under DT4 suggests that dual‐task impairment in MCI may not be limited to frontal cortical alterations (Leone et al. 2017; Pan and Zhang 2025). Rather, it may reflect altered activation patterns across cortical regions involved in attentional allocation, sensory integration, and motor planning. (Papegaaij et al. 2017; Monaghan et al. 2025). Given that successful balance control under dual‐task conditions depends on the involvement of both cognitive‐control and sensorimotor‐related cortical regions, the present findings support the view that MCI is associated with reduced activation across distributed cortical regions when task demands are high (Zhou et al. 2021; Kahya et al. 2022; Kahya et al. 2019).

The choice of verbal fluency as the concurrent cognitive task may also help explain the observed pattern of results. Verbal fluency requires semantic retrieval, executive control, and rapid response generation, and therefore imposes a substantial cognitive load during postural control (Shao et al. 2014). In the present study, the MCI group showed lower VFT performance in DT2 and DT3, indicating that the task was sensitive to cognitive vulnerability under conditions of increased balance demand. Interestingly, no significant between‐group difference in VFT was found in DT4, despite marked differences in balance performance and cortical activation. One possible explanation is that, under the most difficult condition, participants may have prioritized postural safety over cognitive output (Lacour et al. 2008, Yu and Huang 2017). Alternatively, the very high task demand may have compressed cognitive performance overall, thereby reducing the detectability of between‐group differences in VFT scores, as cognitive dual‐task effects do not necessarily increase monotonically with task complexity and may shift toward mutual interference under highly demanding conditions (Marko et al. 2023, Bianchini et al. 2022).

The partial correlation analyses revealed that significant brain–behavior associations were concentrated mainly under the more demanding dual‐task conditions, particularly DT3 and DT4. This pattern suggests that the relationship between cortical recruitment and behavioral performance becomes more apparent when sensory and postural demands increase (Thönnessen et al. 2024). Under relatively simple conditions, participants may rely on relatively preserved control strategies, making the contribution of cortical activation to behavioral variability less detectable (Rubega et al. 2021). However, when task demands approach the limits of postural and attentional control, cortical recruitment may become more closely associated with successful task execution (Fan et al. 2024, Ward et al. 2022).

Importantly, although within‐group partial correlations demonstrated different association patterns between the NC and MCI groups, these findings alone do not establish that the strength of brain–behavior coupling differs between groups. Therefore, we further performed regression‐based interaction analyses to directly examine whether cortical activation‐behavior relationships differed between NC and MCI participants. After correction for multiple comparisons across ROIs, a significant group × activation interaction was identified only for the association between RPFC activation and MLI during DT4. Specifically, the negative association between RPFC activation and mediolateral sway was stronger in the MCI group than in the NC group. This finding suggests that under the eyes‐closed unstable‐support condition, mediolateral postural control in individuals with MCI may depend more strongly on the recruitment of prefrontal resources (Lee et al. 2024).

The absence of significant interaction effects in other brain–behavior associations indicates that the between‐group differences should be interpreted as region‐ and condition‐specific rather than reflecting a widespread alteration of cortical‐behavior coupling (Chen et al. 2022). Nevertheless, the group‐dependent RPFC–MLI relationship observed during DT4 provides additional evidence that MCI may involve altered relationships between cortical activation and postural performance under high‐demand conditions (Lee et al. 2024, Chen et al. 2022). This interpretation is consistent with previous findings showing that prefrontal regions contribute to attentional allocation and postural regulation during challenging balance tasks, while extending previous work by directly testing group differences in brain–behavior coupling (Park and Schott 2025; Kuo et al. 2022).

Several limitations should be considered when interpreting the present findings. First, the cross‐sectional design means that we cannot determine whether these activation patterns predict cognitive decline or conversion to dementia, so the clinical relevance of our balance‐activation correlations remains speculative. Second, although the four dual‐task conditions provided a clear gradient of task difficulty, single‐task cognitive and postural control conditions were not incorporated into the same analytical framework, making it impossible to calculate dual‐task cost directly. Third, the cognitive component was limited to verbal fluency, and the present findings may therefore not generalize to other cognitive processes such as working memory, inhibition, or mental calculation. Fourth, fNIRS primarily measures hemodynamic activity in superficial cortical regions and cannot directly capture the contribution of deeper structures, such as the cerebellum and basal ganglia, which also play important roles in postural control. Finally, although MCI was defined using comprehensive neuropsychological criteria, the group was not further stratified by clinical subtype or biomarker status, and some degree of heterogeneity may therefore have remained.

Despite these limitations, this study provides several important implications. The findings suggest that dual‐task paradigms involving sensory manipulation and unstable support conditions may be more sensitive than simpler conditions for detecting functional vulnerability in older adults with MCI. Moreover, the extension of reduced activation from the prefrontal cortex to sensorimotor‐related regions under the most demanding condition indicates that dual‐task dysfunction in MCI may arise from altered recruitment patterns involving cognitive‐control and sensorimotor‐related cortical regions, rather than from isolated frontal inefficiency. Future studies should adopt longitudinal designs, include larger and more clinically stratified samples, and, when specifically designed to examine factorial interaction effects among cognitive status, visual availability, and support‐surface stability, apply appropriate factorial mixed models or linear mixed‐effects approaches to further characterize these complex relationships. In addition, combining fNIRS with other neuroimaging or neurophysiological techniques may help determine whether these cortical signatures can predict falls, functional decline, or progression of cognitive impairment.

## 5 Conclusion

Older adults with MCI exhibited impaired behavioral performance and reduced cortical activation during cognitive–postural dual‐tasking, particularly under conditions with greater sensory and balance demands. Brain–behavior associations were particularly observed under specific dual‐task conditions, especially the eyes‐closed unstable‐support condition. Although reduced cortical activation was associated with poorer behavioral performance across multiple cortical regions in MCI, regression‐based interaction analysis identified a significant group difference only for the association between RPFC activation and mediolateral postural stability during DT4. These findings indicate that altered brain–behavior coupling in MCI is condition‐ and region‐specific rather than widespread, highlighting selective alteration of the association between prefrontal activation and postural stability under high‐demand dual‐task conditions. High‐demand cognitive–postural dual‐task paradigms combined with fNIRS may provide a sensitive approach for identifying early functional vulnerability in MCI and informing future assessment and intervention strategies.

## Author Contributions

**Chaojie Kan**: conceptualization, funding acquisition, investigation, validation, data curation, visualization, writing – original draft, resources, formal analysis, software. **Shizhe Zhu**: software, validation, project administration, supervision, writing – review and editing. **Qinglei Wang**: methodology, conceptualization, software, validation, writing – review and editing, formal analysis. **Junfan Shen**: investigation, data curation, methodology, software. **Sheng Xu**: methodology, validation, funding acquisition, supervision, resources, project administration, writing – review and editing. **Yuefeng Li**: methodology, validation, funding acquisition, writing – review and editing, project administration, supervision, resources. **Ren Zhuang**: methodology, conceptualization, writing – review and editing, project administration, supervision, resources, funding acquisition. **Chuan Guo**: conceptualization, methodology, data curation, validation, supervision, funding acquisition, project administration, resources, writing – review and editing.

## Funding

This work was supported by the National Key Research and Development Program of China (Grant No. 2023YFC3603604), the Open Project of Jiangsu Key Laboratory of Laboratory Medicine (Grant No. JSKLM‐Z‐2025‐06), and the Changzhou Sci&Tech Program (Grant No. CJ20253139).

## Ethics Statement

All procedures performed in this study were in accordance with the principles of the Declaration of Helsinki. The study was approved by the Ethics Committee of Changzhou Dean Hospital (No. CZDALL‐2025‐002) and registered with the Chinese Clinical Trial Registry (No. ChiCTR2500107603). Written informed consent was obtained from all participants before enrollment.

## Consent

The authors have nothing to report.

## Conflicts of Interest

The authors declare no conflicts of interest.

## Supporting information




**Supplementary Table S1**. Differences of behavioral performance between two groups under dual‐task conditions.
**Supplementary Table S2**. Differences of cortical activation between two groups under dual‐task conditions.
**Supplementary Table S3**. Partial correlation results in the NC group.
**Supplementary Table S4**. Partial correlation results in the MCI group.
**Supplementary Table S5**. Group × cortical activation interaction analyses of brain–behavior associations.

## Data Availability

The datasets used and/or analyzed during the current study are available from the corresponding authors upon reasonable request.
