# Supplementary material for: Brain–Behavior Coupling During Cognitive–Postural Dual‐Tasking in Older Adults With Mild Cognitive Impairment: An fNIRS Study
Source: Brain Behav. 2026 Nov 6;16(11):e71907. doi: 10.1002/brb3.71907 (PMC13741992; doi:10.1002/brb3.71907)
Supplement: Supplementary file 1 [file BRB3-16-e71907-s001.docx]

| **Supplementary Table S1.**  **Differences of behavioral performance between two groups under dual-task conditions** | | | | |
| --- | --- | --- | --- | --- |
|  | NC group (n=27) | MCI group (n=26) | *t*/*Z* | *p* |
| DT1_API | 1.000(0.700, 1.700) | 1.550(0.675, 2.000) | -1.292^a^ | 0.196 |
| DT2_API | 1.000(0.700, 2.000) | 1.700(0.875, 3.025) | -1.997^a^ | **0.046** |
| DT3_API | 1.400(0.900, 1.900) | 2.000(1.175, 2.425) | -2.075^a^ | **0.038** |
| DT4_API | 1.600(1.100, 2.200) | 2.500(1.375, 2.925) | -2.297^a^ | **0.022** |
| DT1_MLI | 0.500(0.300, 1.000) | 0.800(0.500, 1.125) | -1.906^a^ | 0.057 |
| DT2_MLI | 0.700(0.500, 1.700) | 1.450(0.800, 2.300) | -2.304^a^ | **0.021** |
| DT3_MLI | 0.500(0.400, 0.800) | 0.650(0.500, 1.100) | -1.843^a^ | 0.065 |
| DT4_MLI | 0.700(0.500, 1.000) | 1.250(0.775, 1.925) | -2.915^a^ | **0.004** |
| DT1_VFT | 16.000(15.000, 17.000) | 17.000(14.000, 19.000) | -0.485^a^ | 0.628 |
| DT2_VFT | 18.741 ± 3.504 | 15.654 ± 2.576 | 3.643 | **0.001** |
| DT3_VFT | 13.444 ± 2.694 | 11.385 ± 2.801 | 2.729 | **0.009** |
| DT4_VFT | 11.000(10.000, 13.500) | 11.000(9.000, 12.000) | -1.697^a^ | 0.090 |
| a. *Z* value | | | | |

| **Supplementary Table S2.**  **Differences of cortical activation between two groups under dual-task conditions** | | | | | |
| --- | --- | --- | --- | --- | --- |
| ROI | NC group (n=27) | MCI group (n=26) | *t*/*Z* | *p* | *p_adj^b^* |
| DT1_RPFC | 0.015(-0.081, 0.099) | 0.094(-0.036, 0.217) | -1.744^a^ | 0.081 | 0.108 |
| DT1_RS1 | -0.009 ± 0.237 | 0.138 ± 0.250 | -2.204 | 0.032 | 0.096 |
| DT1_RM1 | -0.187 ± 0.223 | 0.112 ± 0.216 | -2.171 | 0.035 | 0.096 |
| DT1_RPMC | -0.031(-0.132, 0.019) | 0.055(-0.126, 0.220) | -1.886^a^ | 0.059 | 0.108 |
| DT1_LPFC | 0.010(-0.133, 0.100) | 0.015(-0.071, 0.161) | -0.890^a^ | 0.374 | 0.374 |
| DT1_LS1 | -0.059(-0.196, 0.116) | 0.098(-0.009, 0.195) | -2.099^a^ | 0.036 | 0.096 |
| DT1_LM1 | -0.012 ± 0.235 | 0.109 ± 0.243 | -1.842 | 0.071 | 0.108 |
| DT1_LPMC | -0.035 ± 0.226 | 0.049 ± 0.199 | -1.442 | 0.155 | 0.177 |
| DT2_RPFC | 0.012(-0.048, 0.098) | -0.057(-0.344, 0.050) | -1.904^a^ | 0.057 | 0.098 |
| DT2_RS1 | -0.016 ± 0.275 | -0.100 ± 0.219 | 1.214 | 0.230 | 0.263 |
| DT2_RM1 | 0.002 ± 0.208 | -0.080 ± 0.182 | 1.532 | 0.132 | 0.176 |
| DT2_RPMC | 0.024 ± 0.225 | -0.095 ± 0.219 | 1.952 | 0.056 | 0.098 |
| DT2_LPFC | -0.004 ± 0.188 | -0.138 ± 0.201 | 2.493 | 0.016 | 0.098 |
| DT2_LS1 | -0.039 ± 0.199 | -0.060 ± 0.135 | 0.448 | 0.656 | 0.656 |
| DT2_LM1 | -0.011 ± 0.162 | -0.101 ± 0.180 | 1.913 | 0.061 | 0.098 |
| DT2_LPMC | -0.007 ± 0.189 | -0.122 ± 0.178 | 2.271 | 0.027 | 0.098 |
| DT3_RPFC | 0.026(-0.065, 0.157) | -0.128(-0.292, 0.043) | -2.936^a^ | 0.003 | **0.012** |
| DT3_RS1 | 0.009(-0.131, 0.089) | -0.134(-0.326, 0.010) | -1.441^a^ | 0.150 | 0.200 |
| DT3_RM1 | 0.013 ± 0.234 | -0.097 ± 0.183 | 1.893 | 0.064 | 0.128 |
| DT3_RPMC | -0.041(-0.144, 0.113) | -0.101(-0.240, -0.005) | -2.313^a^ | 0.021 | 0.056 |
| DT3_LPFC | 0.046 ± 0.226 | -0.143 ± 0.210 | 3.173 | 0.003 | **0.012** |
| DT3_LS1 | 0.005(-0.129, 0.052) | -0.051(-0.268, 0.041) | -1.299^a^ | 0.194 | 0.222 |
| DT3_LM1 | -0.026 ± 0.181 | -0.078 ± 0.180 | 1.044 | 0.301 | 0.301 |
| DT3_LPMC | -0.162 ± 0.221 | -0.097 ± 0.160 | 1.508 | 0.138 | 0.200 |
| DT4_RPFC | 0.039 ± 0.203 | -0.180 ± 0.209 | 3.888 | <0.001 | **0.008** |
| DT4_RS1 | 0.020 ± 0.244 | -0.179 ± 0.237 | 3.016 | 0.004 | **0.011** |
| DT4_RM1 | -0.054 ± 0.184 | -0.181 ± 0.199 | 2.404 | 0.020 | **0.027** |
| DT4_RPMC | 0.023(-0.145, 0.108) | -0.126(-0.314, -0.051) | -2.900^a^ | 0.004 | **0.011** |
| DT4_LPFC | -0.002(-0.146, 0.124) | -0.136(-0.406, -0.023) | -2.669^a^ | 0.008 | **0.013** |
| DT4_LS1 | -0.036 ± 0.234 | -0.163 ± 0.253 | 1.890 | 0.064 | 0.073 |
| DT4_LM1 | -0.044 ± 0.234 | -0.213 ± 0.200 | 2.825 | 0.007 | **0.013** |
| DT4_LPMC | -0.094(-0.252,0.064) | -0.193(-0.348, -0.058) | -1.744^a^ | 0.081 | 0.081 |
| a. *Z* value. b. FDR adjusted *p* values. | | | | | |

| **Supplementary Table S3.**  **Partial correlation results in the NC group** | | | | | | | | | | | | | |
| --- | --- | --- | --- | --- | --- | --- | --- | --- | --- | --- | --- | --- | --- |
| NC | | DT1 | | | DT2 | | | DT3 | | | DT4 | | |
|  |  | API | MLI | VFT | API | MLI | VFT | API | MLI | VFT | API | MLI | VFT |
| RPFC | *r* | 0.203 | 0.439 | -0.080 | 0.350 | 0.231 | -0.020 | -0.458 | -0.107 | 0.528 | -0.060 | -0.738 | 0.203 |
|  | *p* | 0.331 | 0.028 | 0.705 | 0.087 | 0.267 | 0.923 | 0.021 | 0.612 | 0.007 | 0.777 | <0.001 | 0.342 |
|  | *p_adj^a^* | 0.458 | 0.214 | 0.964 | 0.248 | 0.678 | 0.923 | **0.035** | 0.612 | **0.028** | 0.777 | **<0.001** | 0.456 |
| RS1 | *r* | 0.417 | 0.336 | -0.062 | 0.304 | 0.080 | 0.061 | -0.497 | -0.212 | 0.385 | -0.098 | -0.385 | 0.069 |
|  | *p* | 0.043 | 0.100 | 0.768 | 0.139 | 0.702 | 0.776 | 0.012 | 0.308 | 0.057 | 0.641 | 0.058 | 0.748 |
|  | *p_adj^a^* | 0.172 | 0.214 | 0.964 | 0.248 | 0.802 | 0.923 | **0.035** | 0.352 | 0.115 | 0.733 | 0.077 | 0.748 |
| RM1 | *r* | 0.349 | 0.363 | 0.057 | 0.318 | 0.199 | -0.037 | -0.338 | -0.372 | 0.384 | -0.116 | -0.324 | -0.202 |
|  | *p* | 0.095 | 0.074 | 0.786 | 0.122 | 0.339 | 0.865 | 0.106 | 0.067 | 0.064 | 0.580 | 0.115 | 0.343 |
|  | *p_adj^a^* | 0.253 | 0.214 | 0.964 | 0.248 | 0.678 | 0.923 | 0.106 | 0.107 | 0.115 | 0.733 | 0.115 | 0.456 |
| RPMC | *r* | 0.197 | 0.281 | -0.009 | 0.345 | 0.213 | 0.182 | -0.436 | -0.385 | 0.246 | -0.304 | -0.333 | -0.283 |
|  | *p* | 0.346 | 0.173 | 0.964 | 0.091 | 0.306 | 0.394 | 0.029 | 0.057 | 0.237 | 0.140 | 0.104 | 0.171 |
|  | *p_adj^a^* | 0.458 | 0.231 | 0.964 | 0.248 | 0.678 | 0.923 | **0.039** | 0.107 | 0.271 | 0.372 | 0.115 | 0.456 |
| LPFC | *r* | 0.156 | 0.330 | 0.019 | 0.204 | 0.140 | 0.077 | -0.618 | -0.382 | 0.588 | -0.204 | -0.518 | -0.247 |
|  | *p* | 0.458 | 0.107 | 0.928 | 0.327 | 0.503 | 0.722 | 0.001 | 0.060 | 0.002 | 0.327 | 0.008 | 0.234 |
|  | *p_adj^a^* | 0.458 | 0.214 | 0.964 | 0.374 | 0.695 | 0.923 | **0.008** | 0.107 | **0.016** | 0.523 | **0.021** | 0.456 |
| LS1 | *r* | 0.174 | 0.182 | -0.099 | 0.229 | 0.135 | 0.160 | -0.456 | -0.309 | 0.366 | -0.356 | -0.452 | -0.285 |
|  | *p* | 0.405 | 0.384 | 0.639 | 0.270 | 0.521 | 0.454 | 0.022 | 0.133 | 0.072 | 0.081 | 0.023 | 0.177 |
|  | *p_adj^a^* | 0.458 | 0.384 | 0.964 | 0.360 | 0.695 | 0.923 | **0.035** | 0.177 | 0.115 | 0.324 | **0.037** | 0.456 |
| LM1 | *r* | 0.452 | 0.303 | -0.072 | 0.293 | 0.280 | 0.198 | -0.464 | -0.376 | 0.333 | -0.273 | -0.483 | -0.303 |
|  | *p* | 0.026 | 0.141 | 0.731 | 0.155 | 0.175 | 0.353 | 0.022 | 0.064 | 0.112 | 0.186 | 0.015 | 0.150 |
|  | *p_adj^a^* | 0.172 | 0.226 | 0.964 | 0.248 | 0.678 | 0.923 | **0.035** | 0.107 | 0.149 | 0.372 | **0.030** | 0.456 |
| LPMC | *r* | 0.268 | 0.202 | -0.030 | 0.109 | 0.024 | 0.145 | -0.372 | -0.479 | 0.131 | -0.360 | -0.550 | -0.176 |
|  | *p* | 0.205 | 0.333 | 0.888 | 0.603 | 0.911 | 0.500 | 0.073 | 0.015 | 0.542 | 0.077 | 0.004 | 0.399 |
|  | *p_adj^a^* | 0.410 | 0.381 | 0.964 | 0.603 | 0.911 | 0.923 | 0.083 | 0.107 | 0.542 | 0.324 | **0.016** | 0.456 |
| a. FDR adjusted *p* values. | | | | | | | | | | | | | |

| **Supplementary Table S4.**  **Partial correlation results in the MCI group** | | | | | | | | | | | | | |
| --- | --- | --- | --- | --- | --- | --- | --- | --- | --- | --- | --- | --- | --- |
| MCI | | DT1 | | | DT2 | | | DT3 | | | DT4 | | |
|  |  | API | MLI | VFT | API | MLI | VFT | API | MLI | VFT | API | MLI | VFT |
| RPFC | *r* | 0.140 | 0.214 | 0.016 | 0.132 | -0.075 | -0.009 | 0.285 | 0.026 | 0.602 | -0.661 | -0.935 | -0.202 |
|  | *p* | 0.515 | 0.315 | 0.942 | 0.537 | 0.727 | 0.966 | 0.178 | 0.902 | 0.002 | <0.001 | <0.001 | 0.343 |
|  | *p_adj^a^* | 0.801 | 0.924 | 0.942 | 0.779 | 0.865 | 0.966 | 0.237 | 0.913 | **0.008** | **0.002** | **<0.001** | 0.501 |
| RS1 | *r* | 0.108 | 0.151 | 0.210 | 0.023 | 0.037 | -0.409 | 0.482 | 0.117 | 0.444 | -0.250 | -0.367 | -0.058 |
|  | *p* | 0.615 | 0.482 | 0.324 | 0.914 | 0.865 | 0.047 | 0.017 | 0.587 | 0.030 | 0.239 | 0.078 | 0.788 |
|  | *p_adj^a^* | 0.801 | 0.924 | 0.864 | 0.914 | 0.865 | 0.188 | 0.100 | 0.783 | 0.080 | 0.239 | 0.078 | 0.788 |
| RM1 | *r* | 0.321 | 0.118 | -0.016 | -0.252 | -0.173 | -0.322 | 0.407 | 0.203 | 0.310 | -0.404 | -0.635 | -0.115 |
|  | *p* | 0.127 | 0.582 | 0.942 | 0.236 | 0.420 | 0.125 | 0.048 | 0.342 | 0.140 | 0.050 | 0.001 | 0.594 |
|  | *p_adj^a^* | 0.801 | 0.924 | 0.942 | 0.779 | 0.865 | 0.200 | 0.100 | 0.783 | 0.140 | 0.057 | **0.002** | 0.679 |
| RPMC | *r* | 0.083 | 0.085 | 0.094 | -0.176 | -0.157 | -0.233 | 0.239 | 0.133 | 0.313 | -0.621 | -0.648 | -0.241 |
|  | *p* | 0.701 | 0.693 | 0.664 | 0.409 | 0.463 | 0.274 | 0.260 | 0.535 | 0.136 | 0.001 | 0.001 | 0.257 |
|  | *p_adj^a^* | 0.801 | 0.924 | 0.942 | 0.779 | 0.865 | 0.365 | 0.275 | 0.783 | 0.140 | **0.003** | **0.002** | 0.501 |
| LPFC | *r* | 0.135 | 0.305 | 0.073 | -0.026 | -0.049 | -0.373 | 0.414 | 0.289 | 0.607 | -0.722 | -0.897 | -0.189 |
|  | *p* | 0.530 | 0.148 | 0.734 | 0.902 | 0.820 | 0.073 | 0.045 | 0.171 | 0.002 | <0.001 | <0.001 | 0.376 |
|  | *p_adj^a^* | 0.801 | 0.924 | 0.942 | 0.914 | 0.865 | 0.195 | 0.100 | 0.684 | **0.008** | **<0.001** | **<0.001** | 0.501 |
| LS1 | *r* | -0.024 | 0.118 | 0.319 | 0.255 | 0.132 | -0.544 | 0.232 | 0.023 | 0.410 | -0.437 | -0.462 | -0.376 |
|  | *p* | 0.913 | 0.583 | 0.128 | 0.230 | 0.539 | 0.006 | 0.275 | 0.913 | 0.046 | 0.033 | 0.023 | 0.070 |
|  | *p_adj^a^* | 0.913 | 0.924 | 0.864 | 0.779 | 0.865 | **0.048** | 0.275 | 0.913 | 0.092 | **0.044** | **0.026** | 0.280 |
| LM1 | *r* | 0.089 | 0.015 | 0.245 | -0.118 | 0.068 | -0.207 | 0.404 | 0.293 | 0.339 | -0.597 | -0.679 | -0.429 |
|  | *p* | 0.680 | 0.943 | 0.249 | 0.584 | 0.751 | 0.331 | 0.050 | 0.165 | 0.105 | 0.002 | <0.001 | 0.036 |
|  | *p_adj^a^* | 0.801 | 0.943 | 0.864 | 0.779 | 0.865 | 0.378 | 0.100 | 0.684 | 0.140 | **0.004** | **<0.001** | 0.280 |
| LPMC | *r* | 0.229 | 0.025 | 0.028 | -0.147 | 0.125 | -0.323 | 0.287 | 0.145 | 0.326 | -0.442 | -0.478 | -0.244 |
|  | *p* | 0.281 | 0.907 | 0.898 | 0.493 | 0.561 | 0.124 | 0.173 | 0.500 | 0.119 | 0.031 | 0.018 | 0.250 |
|  | *p_adj^a^* | 0.801 | 0.943 | 0.942 | 0.779 | 0.865 | 0.200 | 0.237 | 0.783 | 0.140 | **0.044** | **0.024** | 0.501 |
| a. FDR adjusted *p* values. | | | | | | | | | | | | | |

| **Supplementary Table S5.**  **Group × cortical activation interaction analyses of brain-behavior associations** | | | | | | | |
| --- | --- | --- | --- | --- | --- | --- | --- |
| **Behavioral measure** | **ROI** | **Interaction β** | **SE** | ***t*** | **95% CI** | ***p*** | ***p_adj^a^*** |
| DT4-API | DT4-RPFC | -3.077 | 1.106 | -2.782 | [-5.302, -0.852] | 0.008 | 0.062 |
| DT4-API | DT4-LM1 | -1.842 | 1.164 | -1.583 | [-4.183, 0.499] | 0.120 | 0.481 |
| DT4-API | DT4-LPFC | -1.485 | 1.176 | -1.263 | [-3.851, 0.880] | 0.2123 | 0.567 |
| DT4-API | DT4-RPMC | -1.013 | 1.146 | -0.883 | [-3.319, 1.293] | 0.382 | 0.651 |
| DT4-API | DT4-LPMC | -0.902 | 1.155 | -0.785 | [-3.230, 1.426] | 0.437 | 0.651 |
| DT4-API | DT4-RM1 | -0.970 | 1.115 | -0.699 | [-3.220, 1.280] | 0.488 | 0.651 |
| DT4-API | DT4-LS1 | -0.575 | 1.024 | -0.563 | [-2.642, 1.492] | 0.576 | 0.658 |
| DT4-API | DT4-RS1 | -0.445 | 1.095 | -0.408 | [-2.655, 1.765] | 0.686 | 0.686 |
| **DT4-MLI** | **DT4-RPFC** | **-3.156** | **0.694** | -4.547 | **[-4.553, -1.760]** | <0.001 | **<0.001** |
| DT4-MLI | DT4-LM1 | -1.795 | 1.094 | -1.642 | [-3.996, 0.405] | 0.107 | 0.429 |
| DT4-MLI | DT4-LPFC | -1.091 | 0.925 | -1.180 | [-2.953, 0.770] | 0.244 | 0.442 |
| DT4-MLI | DT4-LPMC | -1.110 | 1.008 | -1.103 | [-3.135, 0.915] | 0.276 | 0.442 |
| DT4-MLI | DT4-RPMC | -1.102 | 1.093 | -1.008 | [-3.302, 1.098] | 0.319 | 0.442 |
| DT4-MLI | DT4-RM1 | -1.223 | 1.248 | -0.980 | [-3.734, 1.287] | 0.332 | 0.442 |
| DT4-MLI | DT4-LS1 | -0.762 | 0.941 | -0.810 | [-2.655, 1.131] | 0.422 | 0.442 |
| DT4-MLI | DT4-RS1 | -0.770 | 0.994 | -0.775 | [-2.770, 1.230] | 0.442 | 0.442 |
| DT3-VFT | DT3-RPFC | 4.359 | 2.914 | 1.496 | [-1.503, 10.220] | 0.141 | 0.799 |
| DT3-VFT | DT3-LPMC | 3.796 | 4.363 | 0.870 | [-4.981, 12.572] | 0.389 | 0.799 |
| DT3-VFT | DT3-LPFC | 1.977 | 3.084 | 0.641 | [-4.227, 8.180] | 0.525 | 0.799 |
| DT3-VFT | DT3-LS1 | 2.036 | 3.318 | 0.613 | [-4.640, 8.711] | 0.543 | 0.799 |
| DT3-VFT | DT3-RS1 | 1.545 | 2.978 | 0.519 | [-4.446, 7.536] | 0.606 | 0.799 |
| DT3-VFT | DT3-RPMC | 1.252 | 4.178 | 0.300 | [-7.152, 9.657] | 0.766 | 0.799 |
| DT3-VFT | DT3-LM1 | 1.184 | 4.308 | 0.275 | [-7.483, 9.851] | 0.785 | 0.799 |
| DT3-VFT | DT3-RM1 | 0.984 | 3.839 | 0.256 | [-6.738, 8.706] | 0.799 | 0.799 |
| a. FDR adjusted *p* values. | | | | | | | |
